# Supplementary material for: Five-year clinical outcomes of 107 consecutive DMEK surgeries
Source: PLoS One. 2023 Dec 21;18(12):e0295434. doi: 10.1371/journal.pone.0295434 (PMC10735023; doi:10.1371/journal.pone.0295434)
Supplement: S1 Fig — Kaplan-Meier analysis of DMEK graft survival probability at 5 years for only the first eyes (n = 80) (A) and only the first-eyes of the FECD patients (n = 74) (B). DMEK, Descemet membrane endothelial keratoplasty; FECD, Fuchs endothelial corneal dystrophy. (DOCX) [file pone.0295434.s001.docx]

## Supplementary Fig S1. Kaplan-Meier analysis of DMEK graft survival probability at 5 years for only the first eyes (n=80) (A) and only the first-eyes of the FECD patients (n=74) (B). DMEK, Descemet membrane endothelial keratoplasty; FECD, Fuchs endothelial corneal dystrophy.


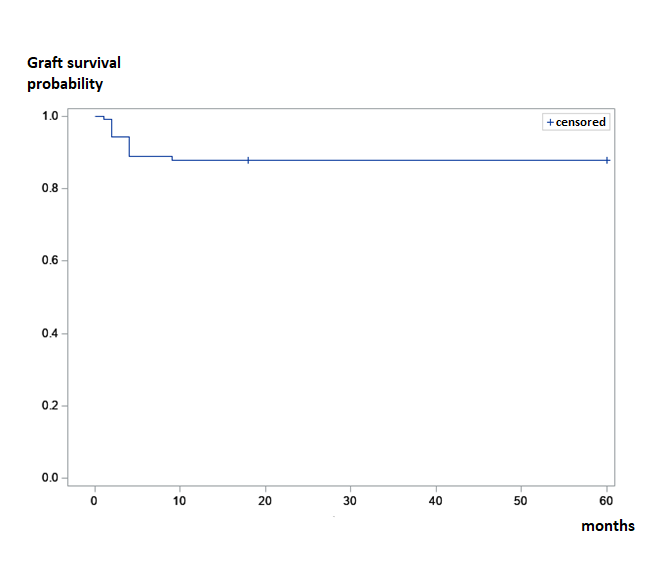


A


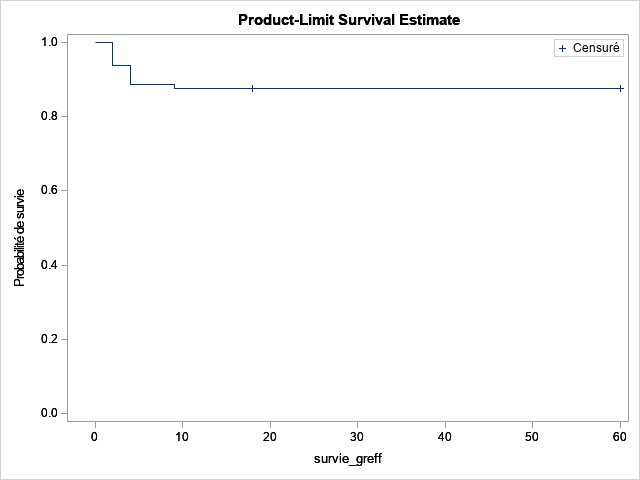


B
