# Supplementary material for: Five-year clinical outcomes of 107 consecutive DMEK surgeries
Source: PLoS One. 2023 Dec 21;18(12):e0295434. doi: 10.1371/journal.pone.0295434 (PMC10735023; doi:10.1371/journal.pone.0295434)
Supplement: S1 Table — (DOCX) [file pone.0295434.s002.docx]

## Supplementary Table S1. Change in BCVA, ECD, and CCT over 5 years after DMEK for the first DMEK eyes (n=80)

| **Variable** | **Preop** | **Month 1** | **Month 3** | **Month 6** | **Year 1** | **Year 2** | **Year 3** | **Year 4** | **Year 5** |
| --- | --- | --- | --- | --- | --- | --- | --- | --- | --- |
| **BCVA**† | *n*=75 | *n*=75 | *n*=69 | *n*=66 | *n*=65 | *n*=65 | *n*=65 | *n*=65 | *n*=65 |
| /10 |  |  |  |  |  |  |  |  |  |
| <5 | 73 (97) | 30 (40) | 12 (17) | 4 (6) | 2 (3) | 2 (3) | 3 (5) | 4 (6) | 6 (9) |
| ≥5 | 2 (3) | 22 (29) | 20 (29) | 15 (23) | 9 (14) | 8 (12) | 6 (9) | 11 (17) | 11 (17) |
| ≥8 | 0 (0) | 13 (17) | 20 (29) | 23 (35) | 25 (38) | 21 (32) | 25 (38) | 18 (28) | 16 (25) |
| ≥10 | 0 (0) | 9 (12) | 16 (23) | 21 (32) | 20 (31) | 20 (31) | 20 (31) | 21 (32) | 23 (35) |
| ≥12 | 0 (0) | 1 (1) | 1 (1) | 3 (5) | 9 (14) | 14 (22) | 11 (17) | 11 (17) | 9 (14) |
| Decimal | 2 (1; 3) | 5 (3; 8) | 8 (6; 9) | 9 (7; 10) | 9 (8; 10) | 10 (8; 10) | 9 (8; 10) | 9 (8; 10) | 9 (7; 10) |
| logMAR | 0.7 (0.5; 1) | 0.3 (0.1; 0.5)* | 0.1 (0.05; 0.2)* | 0.05 (0; 0.15)* | 0.05 (0; 0.1)* | 0 (0; 0.1)* | 0.05 (0; 0.1)* | 0.05 (0; 0.1)* | 0.05 (0; 0.15)* |
| **ECD** | *n*=80 | - | - | *n*=70 | *n*=69 | *n*=69 | *n*=69 | *n*=68 | *n*=68 |
| Cells/mm^2^ | 2560 (2426;2725) | - | - | 1350 (1026;1700)* | 1215 (1000;1550)* | 1100 (825;1400)* | 1009 (800;1309)* | 1000 (785;1261)* | 900 (706;1205)* |
| ECL‡ | - | - | - | -47 (-60;-35) | -53 (-63;-40) | -58 (-68;-45) | -60 (-69;-50) | -61 (-71;-50) | -65 (-72;-53) |
| **CCT** | *n*=71 | - | *n*=8 | *n*=15 | *n*=24 | *n*=33 | *n*=43 | *n*=49 | *n*=53 |
| µm | 620 (580;649) | - | 541 (539;560)* | 540 (535;560)* | 536 (530;554)* | 535 (515;548)* | 542 (518;576)* | 548 (525;573)* | 550 (524;576)* |
| Change§ | - | - | -17 (-19;-14) | -15 (-19;-13) | -14 (-19;-11) | -14 (-18;-8) | -13 (-18;-6) | -11 (-18;-6) | -11 (-17;-5) |

Data are shown as *n* (%) or median (IQR).

* Significantly different relative to preoperative values, as determined by Wilcoxon signed rank test followed by Bonferroni correction (all p<0.0001).

† Seven eyes with preoperative conditions that could affect visual function recovery were excluded from the BCVA analysis.

‡ Endothelial cell loss relative to baseline, expressed as %.

§ Change in CCT relative to baseline, expressed as %. Each calculation was relative to the preoperative CCT of the eyes that were avilable at the timepoint being examined.

BCVA, best-corrected visual acuity; CCT, central corneal thickness, DMEK, Descemet-membrane endothelial keratoplasty; ECD, endothelial cell density; ECL, endothelial-cell loss.
