# Supplementary material for: Five-year clinical outcomes of 107 consecutive DMEK surgeries
Source: PLoS One. 2023 Dec 21;18(12):e0295434. doi: 10.1371/journal.pone.0295434 (PMC10735023; doi:10.1371/journal.pone.0295434)
Supplement: S2 Table — (DOCX) [file pone.0295434.s003.docx]

## Supplementary Table S2. Change in BCVA, ECD, and CCT over 5 years after DMEK for FECD first eyes (n=74)

|  | **Preop** | **Month 1** | **Month 3** | **Month 6** | **Year 1** | **Year 2** | **Year 3** | **Year 4** | **Year 5** |
| --- | --- | --- | --- | --- | --- | --- | --- | --- | --- |
| BCVA† | *n*=70 | *n*=70 | *n*=64 | *n*=63 | *n*=62 | *n*=62 | *n*=62 | *n*=62 | *n*=62 |
| /10 |  |  |  |  |  |  |  |  |  |
| <5 | 68 (97) | 27 (39) | 9 (14) | 4 (6) | 2 (3) | 2 (3) | 3 (5) | 4 (6) | 6 (10) |
| ≥5 | 2 (3) | 21 (30) | 20 (31) | 14 (22) | 8 (13) | 7 (11) | 5 (8) | 10 (16) | 9 (15) |
| ≥8 | 0 (0) | 12 (17) | 18 (28) | 21 (33) | 24 (39) | 20 (32) | 24 (39) | 17 (27) | 16 (26) |
| ≥10 | 0 (0) | 9 (13) | 16 (25) | 21 (33) | 19 (31) | 19 (31) | 19 (31) | 20 (32) | 22 (35) |
| ≥12 | 0 (0) | 1 (1) | 1 (2) | 3 (5) | 9 (15) | 14 (23) | 11 (18) | 11 (18) | 9 (15) |
| Decimal | 2 (1; 3) | 5 (3; 8) | 8 (6; 10) | 9 (7; 10) | 9 (8; 10) | 10 (8; 10) | 9 (8; 10) | 9,5 (8; 10) | 9,5 (8; 10) |
| logMAR | 0.7 (0.5; 1)* | 0.3 (0.1; 0.5)* | 0.1 (0; 0.2)* | 0.05 (0; 0.15)* | 0.05 (0; 0.1)* | 0 (0; 0.1)* | 0.05 (0; 0.1)* | 0.025 (0; 0.1) | 0.025 (0; 0.1)* |
| ECD | *n*=74 | - | - | *n*=66 | *n*=66 | *n*=65 | *n*=65 | *n*=64 | *n*=64 |
| Cells/mm² | 2550 (2420;2700) | - | - | 1350 (1050;1700)* | 1217 (900;1569)* | 1110 (850;1435)* | 1027 (806;1320)* | 1003 (785;1285)* | 900 (713;1230)* |
| ECL‡ | - | - | - | -47 (-59;-35) | -53 (-63;-40) | -57 (-66;-45) | -58 (-69;-49) | -61 (-71;-49) | -65 (-72;-53) |
| CCT | *n*=66 | - | *n*=8 | *n*=15 | *n*=24 | *n*=33 | *n*=42 | *n*=48 | *n*=50 |
| µm | 619 (580;649) | - | 541 (539;560)* | 540 (535;560)* | 536 (530;554)* | 535 (515;548)* | 541 (518;570)* | 546 (521;572)* | 550 (520;575)* |
| Change§ | - | - | -17 (-19;-14) | -15 (-19;-13) | -14 (-19;-11) | -14 (-18;-8) | -13 (-18;-6) | -12 (-18;-6) | -11 (-18;-5) |

Data are shown as *n* (%) or median (IQR).

* Significantly different relative to preoperative values, as determined by Wilcoxon signed rank test followed by Bonferroni correction (all p<0.0001).

† Seven eyes with preoperative conditions that could affect visual function recovery were excluded from the BCVA analysis.

‡ Endothelial cell loss relative to baseline, expressed as %.

§ Change in CCT relative to baseline, expressed as %. Each calculation was relative to the preoperative CCT of the eyes that were avilable at the timepoint being examined.

BCVA, best-corrected visual acuity; CCT, central corneal thickness, DMEK, Descemet-membrane endothelial keratoplasty; ECD, endothelial cell density; ECL, endothelial-cell loss.
